# The Behavioural Dysfunction Questionnaire discriminates behavioural variant frontotemporal dementia from Alzheimer’s disease dementia and major depressive disorder

**DOI:** 10.1007/s00415-023-11666-6

**Published:** 2023-03-23

**Authors:** Anna Semenkova, Olivier Piguet, Andreas Johnen, Matthias L. Schroeter, Jannis Godulla, Christoph Linnemann, Markus Mühlhauser, Thomas Sauer, Markus Baumgartner, Sarah Anderl-Straub, Markus Otto, Ansgar Felbecker, Reto W. Kressig, Manfred Berres, Marc Sollberger

**Affiliations:** 1 Memory Clinic, University Department of Geriatric Medicine FELIX PLATTER, Basel, Switzerland; 2 Faculty of Psychology, University of Basel, Basel, Switzerland; 3 School of Psychology and Brain and Mind Centre, The University of Sydney, Sydney, NSW Australia; 4 Clinic for Neurology, Münster University Hospital, Münster, Germany; 5 Clinic for Cognitive Neurology, University Hospital Leipzig, Leipzig, Germany; 6 Max Planck Institute for Human Cognitive and Brain Sciences, Leipzig, Germany; 7 University Psychiatric Clinic, Basel, Switzerland; 8 Memory Clinic Sonnweid, Wetzikon, Switzerland; 9 Department of Neurology, University of Ulm, Ulm, Germany; 10 Department of Neurology, University Hospital Halle, Halle, Germany; 11 Clinic of Neurology und Neurophysiology, Canton Hospital St. Gallen, St. Gallen, Switzerland; 12 Department of Mathematics and Technology, University of Applied Sciences Koblenz, Koblenz, Germany; 13 Department of Neurology, University Hospital Basel, Basel, Switzerland

**Keywords:** Behavioural variant frontotemporal dementia, Alzheimer’s disease, Depressive disorder, Behavioural disorder, Questionnaire

## Abstract

**Background and objectives:**

Early-stage behavioural variant frontotemporal dementia (bvFTD) is often misdiagnosed, highlighting the need for new diagnostic instruments. Based on the revised diagnostic criteria for bvFTD, we developed the Behavioural Dysfunction Questionnaire (BDQ). In this explorative study, we aimed to determine the best scoring and analytical method for the BDQ to discriminate between bvFTD and non-bvFTD patients.

**Materials and methods:**

34 patients with early-stage bvFTD, 56 with early-stage Alzheimer's disease dementia (ADD) and 41 with major depressive disorder (MDD) were recruited. We calculated BDQ-items with or without inclusion of a time criterion: (a) without time criterion, (b) with 10 years’ time criterion (symptom presence less than 10 years), and (c) with 3 years’ time criterion (symptom presentation within the first 3 years). Using these three differently calculated items, we generated six variables, i.e. 3*2 [BDQ-Global Score (BDQ-GS; domains average score); BDQ-Global Domain Score (BDQ-GDS; domains categorical score)]. Then, we performed univariate and bivariate (BDQ-GS and BDQ-GDS combined) ROC analyses.

**Results:**

Models including BDQ-GS, BDQ-GDS or both variables combined discriminated similarly between groups. In contrast, models without time criterion or with 10 years’ time criterion discriminated better than models including variables with 3 years’ time criterion. These models discriminated highly (AUC = 85.98–87.78) between bvFTD and MDD and bvFTD and ADD, respectively.

**Conclusion:**

BDQ-scores without any time criterion discriminated highly between early-stage bvFTD and non-bvFTD groups, which could improve the early diagnosis of bvFTD. With its standardised procedure, the BDQ is also appropriate for repeated assessments.

**Supplementary Information:**

The online version contains supplementary material available at 10.1007/s00415-023-11666-6.

## Introduction

After Alzheimer’s disease dementia (ADD), frontotemporal dementia (FTD) is the second most common younger-onset dementia, with the behavioural variant (bvFTD) as its most frequent clinical syndrome [27, 31, 35]. BvFTD is a neurodegenerative disorder associated with early progressive changes in personality, behaviour, and social interactions [27, 35], often with only mild and nonspecific cognitive deficits in the early stages of the disease [24]. Currently, no biomarkers exist that enable a reliable early diagnosis in sporadic (i.e. non-genetic) bvFTD cases [13, 35]. Thus, diagnoses rely strongly on clinical assessment, in which one of the main diagnostic challenges is the clinical overlap of bvFTD with primary psychiatric disorders (PPD) [9, 25, 32, 36] and neurodegenerative disorders such as behavioural variant Alzheimer’s disease (AD) [21, 23]. Indeed, up to 50% of bvFTD patients are first diagnosed with PPD and vice versa [15, 29], of which major depressive disorder (MDD) is probably the most common misdiagnosis [21, 29, 36]. Similarly, 7–17% of patients diagnosed with bvFTD are found to have AD pathology post-mortem [2, 12].

In 2011, the revised diagnostic criteria for bvFTD were published [26]. These criteria are based on six clinical domains including five behavioural domains (i.e. early behavioural disinhibition; early apathy/inertia; early loss of sympathy/empathy; early perseverative, stereotyped or compulsive/ritualistic behaviour; and hyperorality/dietary changes) and one cognitive domain (primary executive dysfunction). The risk of examiner-biased assessment of these domains seems quite low among bvFTD experts according to LaMarre, et al. [16], who found moderate to high interrater agreement (*κ* = 0.41–0.80) between the six domains. However, the majority of clinicians, who are evaluating these domains, are not bvFTD experts, which increases the likelihood of examiner-biased assessment. Moreover, endorsement or non-endorsement of a behavioural domain is a crude approach, which does not capture the severity of the behavioural disorders. Consequently, the development of an informant questionnaire, which assesses these five behavioural domains in a standardised and quantitative way, is warranted.

To the best of our knowledge, two instruments are currently available to assess bvFTD-specific behavioural disorders, namely the Frontal Behavioral Inventory (FBI) [14] and DAPHNE (named for Disinhibition, Apathy, Perseverations, Hyperorality, Personal neglect and Loss of Empathy) [6]. The FBI, which exists both as an informant interview and as an informant questionnaire, was designed to optimise diagnostic accuracy for the Lund–Manchester criteria for frontotemporal dementia [11], but also includes items based on the authors´ experience [14]. Accordingly, the FBI comprises items such as inattention or incontinence [14] that are not part of the current diagnostic criteria for bvFTD [26].

Unlike the FBI, DAPHNE [6] is based on the five behavioural domains of the diagnostic criteria for bvFTD [26]. It is an informant interview that is composed of 10 items with five possible answer categories, designed as semi-structured propositions similarly to the Clinical Dementia Rating scale [20]. DAPHNE does not reflect completely the structure of the behavioural domains of the bvFTD diagnostic criteria. First, “personal neglect” is an addition to the five behavioural domains. Second, the behavioural domains of DAPHNE are weighted differently, i.e. behavioural disinhibition represents 40%, hyperorality represents 20% and each of the other four domains represent 10% of the ten items. Third, DAPHNE does not consider the time criterion “early” as required by the diagnostic criteria for scoring four of the five behavioural domains [26].

In light of this evidence, we aimed to develop an instrument that would operationalise the diagnostic criteria for bvFTD more precisely. To meet this goal, we operationalised the five bvFTD-behavioural domains according to the examples of behavioural disorders of the consensus paper for bvFTD [26]. We opted for an informant questionnaire rather than an interview instrument to facilitate its use in clinical practice, and named it “Behavioural Dysfunction Questionnaire” (BDQ). Information on the development of the BDQ is provided in the Methods section and Supplementary Material A.

In this study, we administered the BDQ to informants of patients with probable bvFTD [26], probable ADD [18] and major depressive disorder (MDD) [34].

Our aims were (1) to determine the best scoring method for BDQ to discriminate between bvFTD and the other two patient groups, and (2) to compare its discriminatory power with that of the FBI [14], probably the most common inventory for assessing bvFTD-specific behavioural symptoms at present.

## Methods

### Participants

In total, 131 patients were recruited from several Swiss and German medical centres with expertise in early diagnosis of bvFTD, ADD and/or MDD. Thirty-four patients with probable bvFTD [26], 56 patients with either probable ADD with evidence of the AD pathophysiological process (*n* = 49) or probable ADD (*n* = 7) [18] and 41 patients with MDD (i.e. at least moderate depressive episode according to ICD-10 [34]) were recruited. As the BDQ should primarily help discriminating early-stage bvFTD patients from other patient groups, we included only bvFTD and ADD patients with a major neurocognitive disorder at mild stage according to DSM–5 [3]. An additional inclusion criterion for all patients was availability of a reliable informant (> 18 years) who has regular contact with the patient. Specific exclusion criteria for bvFTD and ADD patients were a major neurocognitive disorder at or above moderate stage according to DSM-5, history of severe depressive episode or current depressive episode according to ICD-10 and history of or current major psychiatric disorders according to ICD-10. Specific exclusion criteria for MDD patients were a neurocognitive disorder according to DSM-5 and any other major psychiatric disorders according to ICD-10. Exclusion criteria for all patient groups were history of or current drug or/and alcohol abuse as well as drug- or/and alcohol-related disorders according to ICD-10 and traumatic brain injuries, systemic disorders or brain diseases that could result in behavioural changes.

To increase diagnostic certainty, we confirmed the majority of patients’ diagnoses [86% (88% bvFTD, 88% ADD (88% probable ADD with evidence of the AD pathophysiological process, 86% probable ADD), 83% MDD)] by at least one follow-up assessment either in the institution or by a standardised phone interview (mean time period of 24 ± 11 months).

As some BDQ-items such as cursing are to some degree not necessarily pathological, we also collected BDQ-data of 414 cognitively and mentally healthy Central European individuals [52% women; age 69.21 ± 12.53 years; 14.61 ± 3.16 years of education] to determine the range of behaviour as measured by the BDQ in the general population. Inclusion and exclusion criteria for the healthy participants are outlined in Supplementary Material B.

### Instruments

#### Behavioural Dysfunction Questionnaire (BDQ)

The BDQ is an informant questionnaire based on the five behavioural domains of the bvFTD diagnostic criteria [26]. Items of each behavioural domain are scored for their frequency or severity on a Likert-scale from 0 (none) to 5 (very often/very severe). For each endorsed item, the informant was also required to state its time of onset. To know whether endorsed items fulfil the time criterion “early” as required by Rascovsky, et al. [26], informants were asked to state both, the time of onset of the endorsed item and the time when the first symptoms appeared. The development and design of the BDQ are presented in Supplementary Material A.

#### Frontal Behavioral Inventory (FBI)

To test the convergent validity of the BDQ, we administered the German version of the FBI as an informant questionnaire [14]. It contains 24 items scored on a Likert-scale from 0 (none) to 3 (severe/most of the time). The total score is the sum of all items.

### Statistical approach

#### Evaluating the need for adjustment of patients’ scores in relation to healthy subjects’ scores

First, we compared healthy subjects’ item scores (Supplementary Material C) with bvFTD patients’ item scores using Kendall-Tau [1] and adjusted patients’ items scores, based on these coefficients. Comparisons between the adjusted and not adjusted patients’ items scores using ROC analyses revealed no significant differences between these scores. As such, all subsequent analyses use unadjusted scores.

#### Exclusion of BDQ-items affirmed by less than 5% of bvFTD patients’ informants

To omit behavioural items not related to early-stage bvFTD, we excluded items endorsed by fewer than 5% of bvFTD patients’ informants. In doing so, we removed 6 of the 56 items (see removed items in Supplementary Material A).

#### Scoring of item scores

Next, we devised three different item scoring methods (a–c):

- Original item scores (i.e. scores unchanged);
- Item scores adjusted according to the 10 years’ time criterion. By assuming that a patient’s behaviour that exists over 10 years is rather a personality trait than due to a neurodegenerative disease or another brain disease, we set any endorsed items with a duration longer than 10 years to zero;
- Item scores of four bvFTD domains (i.e. disinhibition, apathy/inertia, loss of empathy, stereotypic behaviour) were adjusted for time criterion “early” as defined by the diagnostic criteria for bvFTD [26]. “Early” refers to symptom presentation within the first 3 years [26]. Endorsed items that did not fulfil this criterion were set to zero.

#### Generating two global BDQ-scores

BDQ-Global Score (BDQ-GS) was calculated as an average score of the mean domains´ scores. By taking this approach, we ensured that each domain score contributed equally to the total score.

BDQ-Global Domain Score (BDQ-GDS) represents the number of endorsed behavioural domains (0–5). According to the diagnostic criteria, a behavioural domain is endorsed if at least one behavioural feature (i.e. item) of this domain is “persistent or recurrent, rather than single or rare …” [26]. Accordingly, we considered a domain as endorsed if an item was scored as “sometimes/moderate” or greater. As several items were also endorsed in healthy participants, we added “and above healthy subjects’ 99th percentile of this item” as an additional criterion.

By applying the above-mentioned three different scoring methods on these two global scores, we generated six variables:

- 1a. BDQ-GS without time criterion
- 1b. BDQ-GS with 10 years’ time criterion
- 1c. BDQ-GS with 3 years’ time criterion
- 2a. BDQ-GDS without time criterion
- 2b. BDQ-GDS with 10 years’ time criterion
- 2c. BDQ-GDS with 3 years’ time criterion

#### Data analysis

First, we derived a non-bvFTD group by combining ADD and MDD patients, in order to compare the discriminatory power of the six variables between bvFTD and non-bvFTD patients. To test if the merging of these two patient groups is statistically meaningful, we compared the “BDQ-GS without time criterion” score between the ADD and MDD groups. A Kruskal–Wallis test, followed by pairwise Wilcoxon tests, revealed no statistically significant group differences (data not shown), allowing us to combine these participants into a non-bvFTD group.

Next, we run six univariate logistic regressions, followed by ROC analyses. As the BDQ-GS and the BDQ-GDS represent the data differently (i.e. BDQ-GS represents all items independent of domain structure, BDQ-GDS represents number of endorsed domains), we run as well three bivariate (i.e. variables 1a and 2a, variables 1b and 2b, and variables 1c and 2c) logistic regressions in an effort to best separate the groups. Using the Delong’s method [8], we compared the discriminatory power of these nine regression models. We aimed to select the regression model with the highest discriminatory power between bvFTD and non-bvFTD patients. Finally, by taking the best regression model, we aimed (1) to examine its discriminatory power between bvFTD and ADD and between bvFTD and MDD, respectively, and (2) to compare its discriminatory power with the FBI score.

## Results

Analyses of covariance followed by Tukey–Kramer post hoc analyses for age and education and Chi-square test for sex showed no differences among patient groups (Table 1). BvFTD and ADD patients were cognitively more impaired than MDD patients as measured by the Montreal Cognitive Assessment [22]. In addition, bvFTD patients showed higher FBI scores than ADD and MDD patients (Table 1). Based on the Frontotemporal Dementia Rating Scale [19], an informant questionnaire, bvFTD patients experienced on average moderate to severe functional dependence and behavioural disturbances (Table 1).

**Table 1:** Demographic and clinical characteristics of study participants (*N* = 131) classified by diagnostic group

	bvFTD (*n* = 34)	ADD (*n* = 56)	MDD (*n* = 41)	Test (*df*)	Post hoc
Age (years)	64.76 ± 9.78	67.68 ± 10.97	63.32 ± 10.40	2.17_(2, 128)_^a^	
Sex (m/f)	20/14	22/34	17/24	3.57_(2)_^b^	
Education (years)	13.88 ± 2.80	13.14 ± 3.53	13.31 ± 3.34	0.53_(2, 120)_^a^	
MoCA (0–30)	18.6 ± 5.49	17.82 ± 5.10	25.21 ± 3.99	37.84_(2)_^c*^	MDD > bvFTD, ADD*
FBI (0–72)	26.69 ± 12.78	13.6 ± 8.77	12 ± 8.52	27.53_(2)_^c*^	bvFTD > ADD, MDD*
FRS (0–100%)	39.41 ± 23.79	n/a	n/a		

*bvFTD* behavioural variant frontotemporal dementia, *MDD* major depressive disorder, *ADD* Alzheimer’s disease dementia, *MoCA* Montreal Cognitive Assessment, *FBI* Frontal Behavioral Inventory, *FRS* Frontotemporal Dementia Rating Scale (0–2 = profound; 3–12 = very severe; 13–40 = severe; 41–79 = moderate; 80–96 = mild; 97–100 = very mild), *n/a* not applicable

*p < 0.001

^a^Analysis of variance

^b^Chi-square test

^c^Kruskal–Wallis test

The internal consistencies of the five BDQ-domains ranged from poor (*α* = 0.54; “hyperorality and dietary changes”), over acceptable (*α* = 0.67; “early apathy/inertia”) to good (*α* = 0.76–0.86; “early behavioural disinhibition”, “early loss of sympathy/empathy” and “early perseverative/stereotyped behaviour”). The BDQ showed an excellent overall internal consistency (*α* = 0.92). Please see Supplementary Material D for more detail.

### Determining the best BDQ scoring method

The nine regression models showed acceptable to excellent [17] discriminatory power between bvFTD and non-bvFTD patients (AUC ranging between 78.08 and 87.78%) (Table 2). Neither models including BDQ-GS variables nor models including BDQ-GDS variables turned out stronger. Likewise, bivariate regression models did not discriminate better than univariate regression models. To determine whether our findings were driven by single domains, we run post hoc univariate regression analyses with each behavioural domain’s mean score and found similar discriminatory accuracies (Supplementary Material E). Despite the fact that both global scores discriminated similarly, we favoured the BDQ-GS over the BDQ-GDS, as the BDQ-GS is more informative, i.e. it considers the degree of each item, whereas the BDQ-GDS is limited to the number of endorsed behavioural domains.

**Table 2:** Area under the curves of six univariate and three bivariate logistic regression models in bvFTD and non-bvFTD patients

	Without time criterion	Ten years’ time criterion	Three years’ time criterion	Delong´s method
BDQ-GS models	85.98 (CI 78.73–93.22)	86.11 (CI 78.43–93.79)	79.52 (CI 69.98–89.05)	Model 1a, model 1b > model 1c*
BDQ-GDS models	86.43 (CI 79.05–93.81)	86.81 (CI 79.26–94.36)	78.08 (CI 68.10–88.05)	Model 2a, model 2b > model 2c**
BDQ-GS & BDQ-GDS models	87.36 (CI 80.18–94.53)	87.78 (CI 80.49–95.07)	79.64 (CI 70.11–89.17)	Model 3a, model 3b > model 3c**

1a. BDQ-GS without time criterion, 1b. BDQ-GS with 10 years’ time criterion, 1c. BDQ-GS with 3 years’ time criterion, 2a. BDQ-GDS without time criterion, 2b. BDQ-GDS with 10 years’ time criterion, 2c. BDQ-GDS with 3 years’ time criterion, 3a. BDQ-GS without time criterion and BDQ-GDS without time criterion, 3b. BDQ-GS with 10 years’ time criterion and BDQ-GDS with 10 years’ time criterion, 3c. BDQ-GS with 3 years’ time criterion and BDQ-GDS with 3 years’ time criterion

*BDQ-GS* BDQ-Global Score, *BDQ-GDS* BDQ-Global Domain Score

*p < 0.1, **p < 0.05

Regarding the time criterion, models that included the variables without time criterion or with 10 years’ time criterion tended to discriminate better than the models that included variables with the 3 years’ time criterion (*p* = 0.02–0.08). Post hoc analyses in patients with follow-up BDQ assessments (*n* = 44; mean time period of 16.5 ± 6.43 months) revealed that informants’ data on symptoms’ onset, on which we based the time criteria, had low reliability. They deviated on average by 14.47 months (0.03–145.33 months) between two time points. Given these findings, plus the fact that the collection of time data turned out to be elaborate, we decided to omit the time criterion in all subsequent analyses.

In the end, we decided to select the variable “BDQ-GS without time criterion” for further analyses.

### Discriminatory power of BDQ between bvFTD and ADD and between bvFTD and MDD, respectively

Similar discriminatory power between bvFTD and ADD (AUC = 87.84%) and between bvFTD and MDD (AUC = 83.43%) was observed based on the “BDQ-GS without time criterion” variable. Applying the Youden-Index, no single cut-off scores with sufficient sensitivity and specificity were identified. Therefore, we decided to identify two cut-off scores, that is, in each analysis, the cut-off scores with at least 90% sensitivity or 90% specificity [10, 30].

**Fig. 1:**
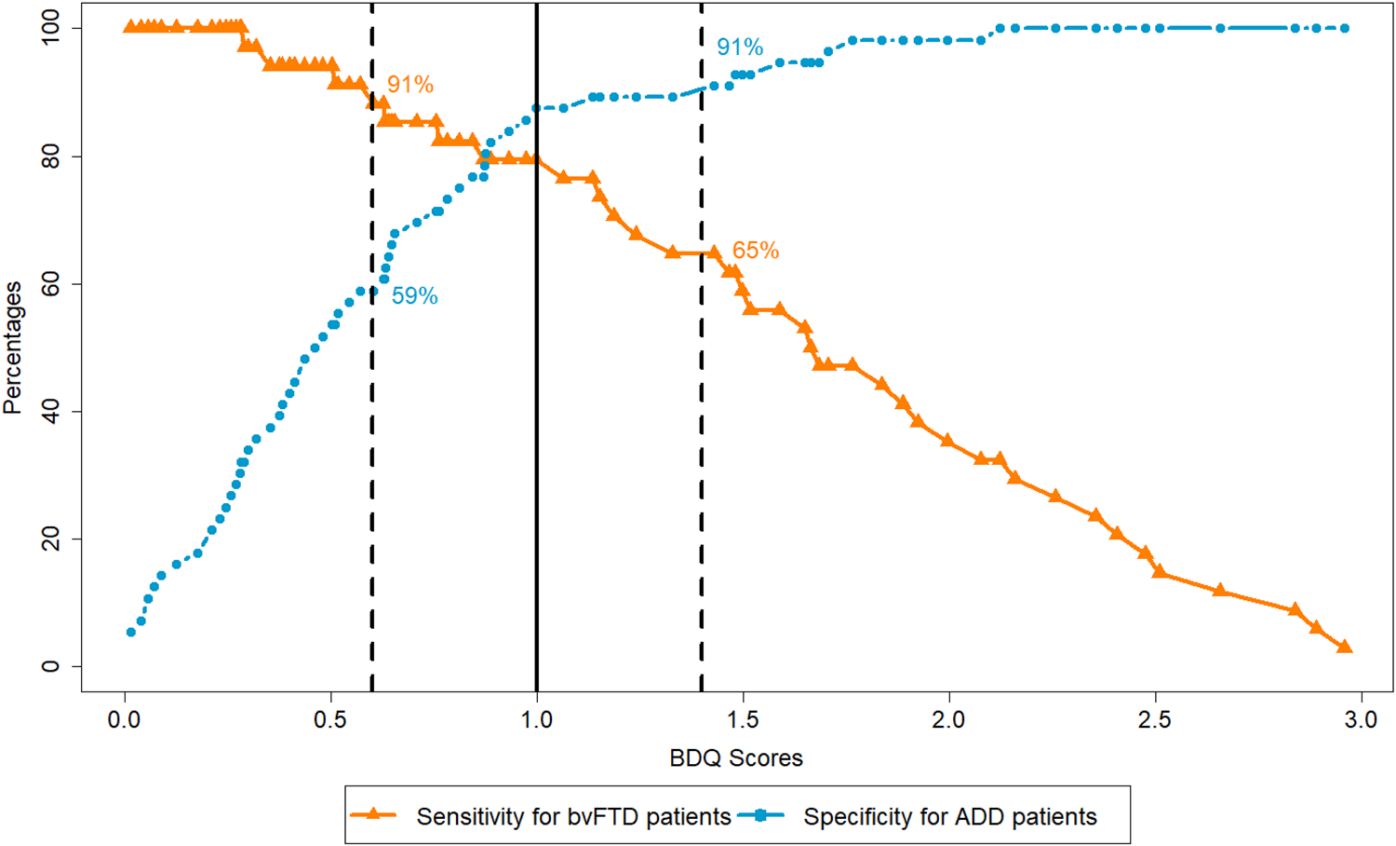
Cut-offs between bvFTD and ADD patients. Percentages of patients with bvFTD who were correctly classified [sensitivity, orange line with triangles] and percentages of correctly classified ADD patients [specificity, blue line with circles] in relation to the BDQ-scores. Two cut-offs with either sensitivity or specificity above 90% are highlighted by dashed lines. The solid black line represents the optimal cut-off using the Youden-Index (sensitivity = 79%; specificity = 88%). *bvFTD* behavioural variant frontotemporal dementia, *ADD* Alzheimer’s disease dementia, *BDQ* Behavioural Dysfunction Questionnaire

When examining the discriminatory power between bvFTD and ADD, we found a score of > 1.4 to be strongly indicative for bvFTD (sensitivity 65%, specificity 91%) and score of < 0.6 to be strongly indicative for ADD (sensitivity 91%, specificity 59%). Scores between 0.6 and 1.4 were considered equivocal (Fig. 1).

**Fig. 2:**
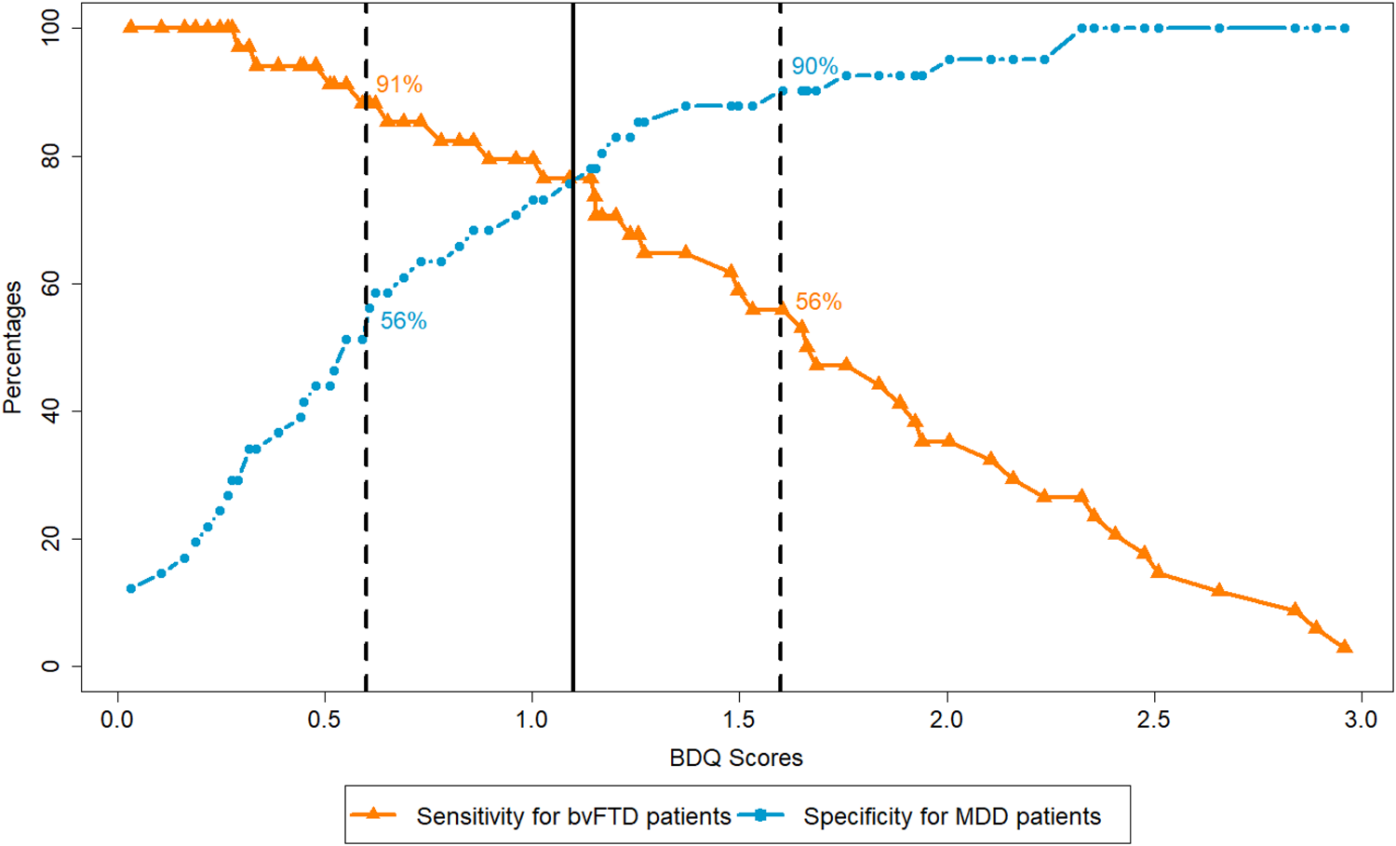
Cut-offs between bvFTD and MDD patients. Percentages of patients with bvFTD who were correctly classified [sensitivity, orange line with triangles] and percentages of correctly classified MDD patients [specificity, blue line with circles] in relation to the BDQ-scores. Two cut-offs with either sensitivity or specificity above 90% are highlighted by dashed lines. The solid black line represents the optimal cut-off using the Youden-Index (sensitivity = 76%; specificity = 78%). *bvFTD* behavioural variant frontotemporal dementia, *MDD* major depressive disorder, *BDQ* Behavioural Dysfunction Questionnaire

When examining the discriminatory power between bvFTD and MDD, we found a score of > 1.6 to be strongly indicative for bvFTD (sensitivity 56%, specificity 90%) and score of < 0.6 to be strongly indicative for MDD (sensitivity 91%, specificity 56%). Scores between 0.6 and 1.6 were considered equivocal (Fig. 2).

Lastly, we compared the discriminatory power of the “BDQ-GS without time criterion” variable with the one of the FBI variable. We found similar discriminatory powers of the two variables between bvFTD and ADD patients, and between bvFTD and MDD patients, respectively.

## Discussion

We developed an informant questionnaire based on the five behavioural domains of the revised diagnostic criteria for bvFTD [26]. This questionnaire, named BDQ, demonstrated excellent [17] discriminatory power between early-stage bvFTD and early-stage ADD patients (AUC = 88%) and between early-stage bvFTD and MDD patients (AUC = 83%), respectively.

### Comparison of two different global scoring methods

We examined whether scoring by a global score (i.e. average score across all domains), by a global domain score (i.e. number of endorsed domains) or by both scores combined would yield different discriminatory powers. These different approaches showed similar results. Given that the global score incorporates all item scores and the global domain score incorporates the number of endorsed domains based on at least one item, these results suggest that items within a domain represent similar behaviours. Indeed, the internal consistencies of the five behavioural domains were acceptable to good apart from the domain “hyperorality and dietary changes” (*α* = 0.54). In addition, given that an overall behavioural measure, independent of domain structure, as measured by the global score separates groups similarly to a behavioural pattern measure, as measured by the global domain score, suggests that each domain discriminated similarly well. Indeed, we found similar discriminatory power of the domain scores between bvFTD and non-bvFTD patients. Taken together, we favoured the global score over the global domain score for further use as it represents a more fine-grained assessment of the different behavioural domains. By contrast, the global domain score is useful for describing the behavioural disorder pattern; information that the global score lacks.

### Investigating whether to include a time criterion in the BDQ scoring

As required by the bvFTD diagnostic criteria [26], we included the time criterion “early” in the BDQ scoring. The time criterion “early” limits scoring to symptoms that appear within the first 3 years in four (i.e. disinhibition, apathy/inertia, loss of empathy and stereotypical behaviour) of the five behavioural domains. As suggested by the diagnostic criteria, we expected that inclusion of this time criterion would increase the discriminatory power between bvFTD and non-bvFTD patients. Inclusion of this strict time criterion, however, resulted in a weaker discriminatory power. Inclusion of a more lenient time criterion, namely limitation of scoring to symptoms that are present for less than 10 years (for removal of any potential personality-associated behavioural abnormalities), did not increase the discriminatory power. In light of these results, we wondered about the reliability of informants' time data on symptoms’ onset and compared informants' time data at two different time points. This analysis revealed large data variability, ranging from 0.03 to 145.33 months. Our findings are consistent with a previous study which showed variance in patients’ recollections of their past symptoms from one inquiry to the next [4]. These findings show the difficulty in perceiving and recalling the time of one’s [4] or another person’s symptom onset, likely even more so if the symptoms develop gradually and affect behaviour. Next to this, the collection of time data turned out to be time-consuming as informants often forgot to state it. Accordingly, we decided to leave out time criteria in future BDQ scoring.

### Comparison of the discriminatory power of the BDQ with existing instruments

The BDQ showed similar discriminatory power to the FBI [14]. At first look, this result seems surprising, since FBI-items represents primarily the Lund–Manchester criteria [11], which should be less sensitive for bvFTD than the current diagnostic criteria for bvFTD [26] upon which the BDQ is based. However, information on the specificity of the revised diagnostic criteria for bvFTD is lacking [26], which limits prediction on their diagnostic accuracy. Moreover, the FBI includes items like “loss of insight” or “personal neglect” that are typical for bvFTD [5, 28], but that are not (i.e. loss of insight), or not prominently (i.e. personal neglect), present in the diagnostic criteria for bvFTD. Nevertheless, the BDQ provides, in contrast to the FBI, not only a global score, but also scores of each behavioural domain, allowing quantitative representation of a patient's pattern of behavioural disturbances. In short, though BDQ and FBI discriminated patient groups similarly well, we consider the BDQ more useful for further use as it has an arranged structure of the behavioural domains and employs the current bvFTD diagnostic criteria [26]. Furthermore, the relatively large number of items lends itself to subsequent data-based weighting of items depending on the comparison group to bvFTD, which would improve the discriminatory power of the BDQ.

The second existing instrument that captures bvFTD-specific behavioural disorders is DAPHNE [6]. In one study, DAPHNE scores discriminated between bvFTD and ADD patients with AUC values between 95 and 99% [6]. These scores are higher than our AUC value (i.e. 88%). However, comparison in discriminatory power between these two instruments should be made with caution as they were used in different samples. Having said this, in the DAPHNE sample, the FBI discriminated between bvFTD and ADD patients similarly well to the DAPHNE [6]. This finding, in turn, corresponds to our finding, i.e. BDQ and FBI separated the two groups in our sample similarly. Accordingly, one may imagine that BDQ and DAPHNE discriminate similarly between bvFTD and ADD patients. The structure of the two instruments, however, differs in that DAPHNE is a semi-structured interview instrument that allows some interaction between informants and examiners, whereas BDQ is a self-administered informant questionnaire that can be completed in the absence of an examiner. Next, although the ten items of DAPHNE are based on the bvFTD diagnostic criteria [26], their compilation and structure is based on French experts’ opinions in bvFTD [6]. Accordingly, unlike the BDQ, DAPHNE does not fully represent the structure of the behavioural domains of the diagnostic criteria for bvFTD. It would be worthwhile to use both instruments in future studies in the same sample to investigate whether they discriminate differently between bvFTD and other patient groups.

### Limitations

Despite the acceptable to excellent discriminatory power of the BDQ between bvFTD and the other two patient groups, a large gap of equivocal results between the two 0.9 sensitivity/specificity thresholds (0.6–1.4 for bvFTD vs. ADD and 0.6–1.6 for bvFTD vs. MDD, respectively) was present. However, it should be noted that the BDQ has only a supportive role in the diagnosis of bvFTD. It records the report of a significant other about a patient’s bvFTD-specific behavioural features in daily life in a standardised way. For the final assessment of these behavioural features, the clinical impression of the examiner on the patient needs to be added. Of course, the assessment of bvFTD-specific behavioural features does not suffice to diagnose bvFTD. A comprehensive clinical assessment plus a brain MRI needs to take place, ideally complemented by further imaging techniques (e.g. FDG-PET, amyloid-PET or tau-PET) and/or laboratory tests (e.g. CSF biomarkers for AD or neurofilament light chain) to increase diagnostic certainty [7, 33].

Our study is limited by the absence of post-mortem pathological confirmations of our patients’ diagnoses. Accordingly, we cannot exclude the possibility of patients’ misdiagnoses. To increase diagnostic certainty, we confirmed the majority of patients’ diagnoses by follow-up assessments (mean time period of 24 ± 11 months).

## Conclusion

In summary, we demonstrated that the BDQ, the first instrument that operationalises the five behavioural domains of the diagnostic criteria for bvFTD [26], discriminates well between bvFTD and two non-bvFTD patient groups. Importantly, it allows a quantitative assessment of these domains that is independent of the examiner’s expertise in bvFTD. This point is significant as knowledge and expertise in bvFTD is generally low outside of research institutions what likely contributes to bvFTD over- and underdiagnoses [21, 29, 36]. With its standardised approach, the BDQ would also be appropriate for assessing the severity of single and all bvFTD-specific behavioural features together. Similarly, it would be also appropriate for follow-up assessments. Last, but not least, the self-administrative format of the BDQ enables time saving behavioural disorder assessment, which is of increasing importance in clinical routine.

## Supplementary Information

Below is the link to the electronic supplementary material.

Supplementary file1 (DOCX 354 kb)

## Data Availability

The datasets used and/or analysed during the current study are available from the corresponding author on reasonable request.
