# Supplementary material for: The Behavioural Dysfunction Questionnaire discriminates behavioural variant frontotemporal dementia from Alzheimer’s disease dementia and major depressive disorder
Source: J Neurol. 2023 Mar 23;270(7):3433–41. doi: 10.1007/s00415-023-11666-6 (PMC10267256; doi:10.1007/s00415-023-11666-6)
Supplement: Supplementary file 1 — Supplementary file1 (DOCX 354 kb) [file 415_2023_11666_MOESM1_ESM.docx]

**Supplementary Material**

**The Behavioural Dysfunction Questionnaire discriminates behavioural variant frontotemporal dementia from Alzheimer’s disease dementia and major depressive disorder**

**Journal of Neurology**

Anna Semenkova^1,2^, Olivier Piguet^3^, Andreas Johnen^4^, Matthias L. Schroeter^5,6^, Jannis Godulla^5,6^, Christoph Linnemann^7^, Markus Mühlhauser^7^, Thomas Sauer^7^, Markus Baumgartner^8^, Sarah Anderl-Straub^9^, Markus Otto^9,10^, Ansgar Felbecker^11^, Reto W. Kressig^1^, Manfred Berres^12^, and Marc Sollberger^1,13^

| *1* | Memory Clinic, University Department of Geriatric Medicine FELIX PLATTER, Basel, Switzerland |
| --- | --- |
| *2* | Faculty of Psychology, University of Basel, Switzerland |
| *3* | The University of Sydney, School of Psychology and Brain and Mind Centre, NSW, Australia |
| *4* | Clinic for Neurology, Münster University Hospital, Münster, Germany |
| *5* | Clinic for Cognitive Neurology, University Hospital Leipzig, Germany |
| *6* | Max Planck Institute for Human Cognitive and Brain Sciences, Leipzig, Germany |
| *7* | University Psychiatric Clinic, Basel, Switzerland |
| *8* | Memory Clinic Sonnweid, Wetzikon, Switzerland |
| *9* | Department of Neurology, University of Ulm, Ulm, Germany |
| *10* | Department of Neurology, University Hospital Halle, Germany |
| *11* | Clinic of Neurology und Neurophysiology, Canton Hospital St. Gallen, Switzerland |
| *12* | Faculty of Mathematics and Technology, University of Applied Sciences Koblenz, Germany |
| *13* | Department of Neurology, University Hospital Basel, Switzerland |

Correspondence concerning this article should be addressed to Marc Sollberger, Memory Clinic, University Department of Geriatric Medicine FELIX PLATTER, Burgfelderstrasse 101, 4055 Basel, Switzerland. Email: marc.sollberger@felixplatter.ch

**Table of content**

[Supplementary A. Development of the Behavioural Dysfunctional Questionnaire 4](#_Toc120545529)

[Supplementary B. Inclusion and exclusion criteria of patients and healthy participants 16](#_Toc120545530)

[Supplementary C. Item scores of the Behavioural Dysfunction Questionnaire of healthy participants 18](#_Toc120545531)

[Supplementary D. Internal consistency of the Behavioural Dysfunctional Questionnaire 21](#_Toc120545532)

[Supplementary E. Discriminatory power of the domain scores and the BDQ-Global Score between bvFTD and Non-bvFTD patients 22](#_Toc120545533)

[References 23](#_Toc120545534)

# Supplementary A. Development of the Behavioural Dysfunctional Questionnaire

The revised diagnostic criteria for behavioural variant frontotemporal dementia (bvFTD), involve six domains with two to three subdomains (Table A1) [7]. The first five domains (A–E) include behavioural disorders, whereas the sixth domain (F) includes a cognitive disorder (i.e., primary executive dysfunction).

**Table A1.** *Domains and subdomains of the diagnostic criteria for behavioural variant frontotemporal dementia*

| **A. Early* behavioural disinhibition** |
| --- |
| A.1. Socially inappropriate behaviour  A.2. Loss of manners or decorum  A.3. Impulsive, rash or careless actions |
| **B. Early* apathy or inertia** |
| B.1. Apathy  B.2. Inertia |
| **C. Early* loss of sympathy or empathy** |
| C.1. Diminished response to other people’s needs and feelings  C.2. Diminished social interest, interrelatedness or personal warmth |
| **D. Early* perseverative, stereotyped or compulsive/ritualistic behaviour** |
| D.1. Simple repetitive movements  D.2. Complex, compulsive or ritualistic behaviours  D.3. Stereotypy of speech |
| **E. Hyperorality and dietary changes** |
| E.1. Altered food preferences  E.2. Binge eating, increased consumption of alcohol or cigarettes  E.3. Oral exploration or consumption of inedible objects |
| **F. Neuropsychological profile: executive/generation deficits with relative sparing of memory and visuospatial functions** |
| F.1. Deficits in executive tasks  F.2. Relative sparing of episodic memory  F.3. Relative sparing of visuospatial skills |

* “early” refers to symptom presentation within the first three years

A domain is considered as affirmed if at least one symptom of this domain is persistent or recurrent [7]. Rascovsky, et al. [7] provided examples of symptoms of each subdomain. Please see for example the subdomain “socially inappropriate behaviour” (Fig. A1).

**Figure A1.** *Exemplary symptoms of the subdomain “socially inappropriate behaviour”*


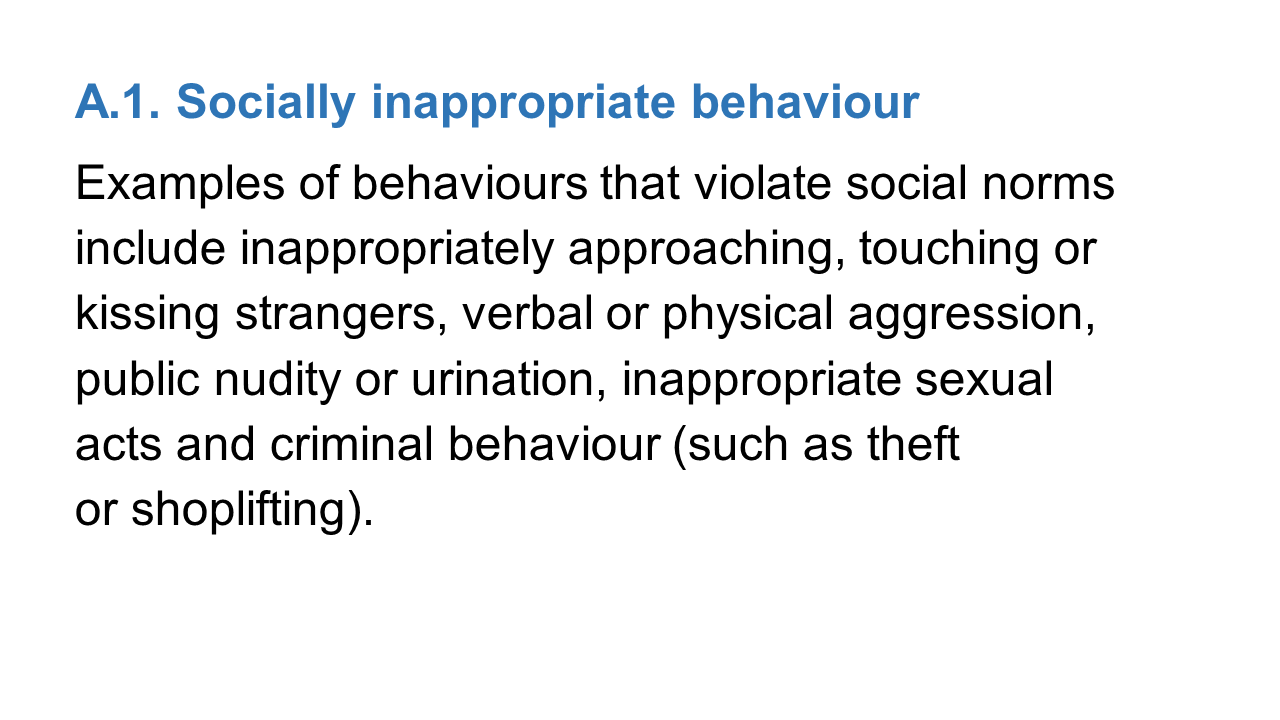


*Development of the questions*

We aimed to operationalise the five behavioural domains of the diagnostic criteria for bvFTD [7] by developing an informant questionnaire. To do this, we kept the structure of the five domains and their subdomains and added the exemplary symptoms from the Appendix of the consensus paper [7] as items to the respective subdomain. First, we translated and retranslated the subdomains and symptoms by native English and German speakers, respectively. Please see, as an example, the subdomain “socially inappropriate behaviour” in English and German language in Table A2.

**Table A2.** *Subdomain “socially inappropriate behaviour” according to Rascovsky, et al. [7] in English and German*

| *A.1. Socially inappropriate behaviour* | *A.1. Sozial unangebrachtes Verhalten* |
| --- | --- |
| Examples of behaviours that violate social norms include inappropriately approaching, touching or kissing strangers, verbal or physical aggression, public nudity or urination, inappropriate sexual acts and criminal behaviour (such as theft or shoplifting). | Beispiele von Verhalten, das soziale Normen verletzt, sind unpassende Annäherungen, Berühren oder Küssen von Fremden, verbale oder körperliche Aggression, öffentliche Nacktheit oder öffentliches Urinieren, unpassende sexuelle Handlungen und kriminelles Verhalten (wie Diebstahl oder Ladendiebstahl). |

Second, we generated questions based on each translated subdomain, e.g., “Zeigt sie/er sozial unangebrachtes Verhalten, wie zum Beispiel …” / “Does she/he show socially inappropriate behaviour, such as …” (Fig. A2, orange colour).

Third, we added the symptoms that belong to the corresponding subdomain and named them “items”. For example, “Unangemessene Annäherungen, wie fremde Personen anfassen oder körperlich ganz nahe kommen” / “Inappropriate approaches, such as touching strangers or getting very close physically ” was named item 1.1 (Fig. A2, blue colour).

**Figure A2.** *Operationalisation of the* *subdomain “socially inappropriate behaviour”*


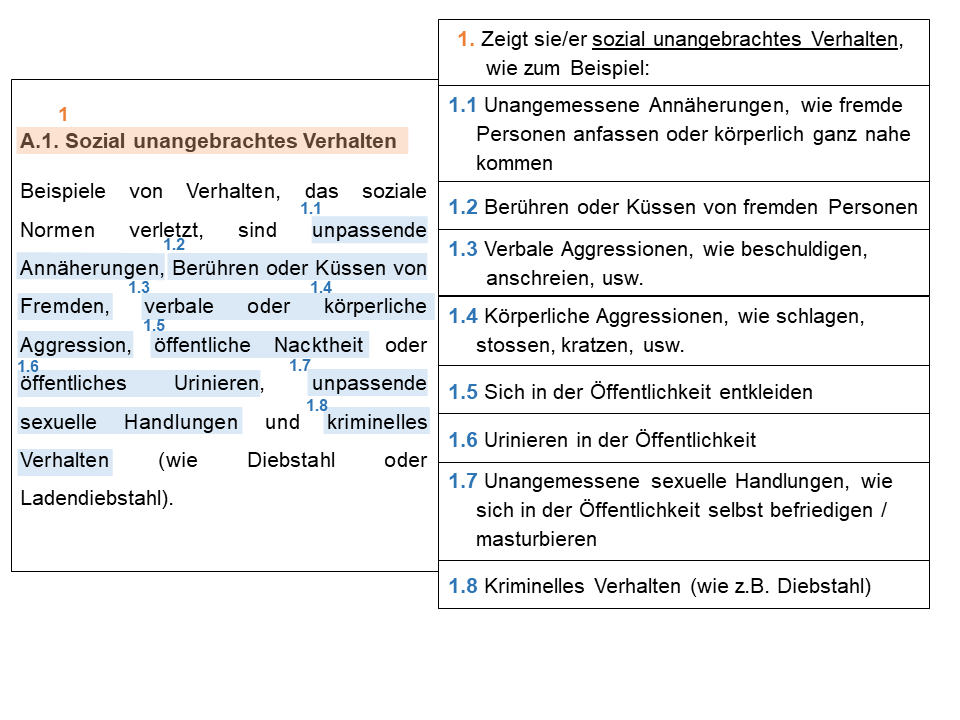


*Generation of item scores*

We decided to use a six-point (0-5) Likert scale to score each item. Similarly to the Frontal Behavioral Inventory (FBI) [4], we decided to use both, severity and frequency, to measure the degree of behavioural disorders, as some disorders are represented best by severity such as apathy or empathy and others best by frequency such as compulsive behaviour. We described each point of the Likert scale as follows

0 – no;

1 – very mild / rare (less than once a month);

2 – mild / occasionally (approximately once a month but not weekly);

3 – moderate / sometimes (about once a week);

4 – severe / often (several times a week but not daily);

5 – very severe / very often (daily)

Four of the five behavioural domains (A-D) are marked with the time criterion “early”. “Early” refers to symptom presentation within the first three years [7]. We operationalized this criterion by asking the informants about both, the respective symptom onset and the clinical onset.

*Examining the clarity of the BDQ and its feasibleness in administration*

To check the BDQ for its clarity and feasibleness in administration, we asked several employees of the Memory Clinic Basel and 20 patients’ caregivers of different ages, sexes and education to read each question carefully for its clarity and to fill out the questionnaire by thinking of a relative’s behaviour. Afterwards, we interviewed them about the clarity of the questionnaire’s instruction, the questions and the answer options. Their comments and suggestions were analysed and applied, if judged as appropriate. In the following, you see the structure of the BDQ using the example of the subdomain “socially inappropriate behaviour” (Table A3).

**Table A3** *Structure of the subdomain “socially inappropriate behaviour”*

| **1.** | **Zeigt sie/er sozial unangebrachtes Verhalten, wie zum Beispiel:** | **NEIN** | **Wenn JA, seit wann?**  (Anzahl Wochen, Monate oder Jahre) | **Aktuelle Häufigkeit/Schweregrad** | | | | |
| --- | --- | --- | --- | --- | --- | --- | --- | --- |
|  |  |  |  | **Sehr selten/ Sehr leicht** (Weniger als 1x/Monat) | **Selten/ Leicht** (Ca. 1x/Monat, aber nicht wöchentlich) | **Manchmal/ Mittel** (Ca. 1x/Woche) | **Häufig/ Stark** (Mehrmals pro Woche, aber nicht täglich) | **Sehr häufig/ Sehr stark** (Mind. 1x/Tag) |
| 1.1 | Unangemessene Annäherungen, wie fremde Personen anfassen oder körperlich ganz nahe kommen | 0 |  | 1 | 2 | 3 | 4 | 5 |
| 1.2 | Berühren oder Küssen von fremden Personen | 0 |  | 1 | 2 | 3 | 4 | 5 |
| 1.3 | Verbale Aggressionen, wie beschuldigen, anschreien, usw. | 0 |  | 1 | 2 | 3 | 4 | 5 |
| 1.4 | Körperliche Aggressionen, wie schlagen, stossen, kratzen, usw. | 0 |  | 1 | 2 | 3 | 4 | 5 |
| 1.5 | Sich in der Öffentlichkeit entkleiden | 0 |  | 1 | 2 | 3 | 4 | 5 |
| 1.6 | Urinieren in der Öffentlichkeit | 0 |  | 1 | 2 | 3 | 4 | 5 |
| 1.7 | Unangemessene sexuelle Handlungen, wie sich in der Öffentlichkeit selbst befriedigen / masturbieren | 0 |  | 1 | 2 | 3 | 4 | 5 |
| 1.8 | Kriminelles Verhalten (wie z.B. Diebstahl) | 0 |  | 1 | 2 | 3 | 4 | 5 |

The final version of BDQ included 56 items corresponding to 15 questions (Table A4). As the number of symptoms differs between subdomains in the diagnostic criteria [7], the number of items between questions differs too. In addition to answering the items, informants had the opportunity to note additional symptoms (question 16). At the end of the questionnaire, the informants were asked about the patient’s first symptom(s) and the clinical onset (question 17).

**Table A4.** *Structure of the Behavioural Dysfunction Questionnaire*

| **Behavioural domains** | **Subdomains** | **Questions** | **Items** |
| --- | --- | --- | --- |
| A. Early* behavioural disinhibition | A1. Socially inappropriate behaviour | 1 | 1.1 – 1.8 |
|  | A2. Loss of manners or decorum | 2 | 2.1 – 2.9 |
|  | A3. Impulsive, rash or careless  actions | 3 | 3.1 – 3.5 |
|  |  |  |  |
| B. Early* apathy or inertia | B1. Apathy | 4 | 4.1 – 4.2 |
|  | B2. Inertia | 5 | 5.1 – 5.2 |
|  |  |  |  |
| C. Early* loss of sympathy or empathy | C1. Diminished response to other people’s needs and feelings | 6 | 6.1 – 6.2 |
|  | C2. Diminished social interest, interrelatedness or personal warmth | 7, 8 | 7.1 – 7.3  8.1 – 8.3 |
|  |  |  |  |
| D. Early* perseverative, stereotyped or compulsive/ritualistic behaviour | D1. Simple repetitive movements | 9 | 9.1 – 9.9 |
|  | D2. Complex, compulsive or ritualistic behaviours | 10 | 10.1 – 10.7 |
|  | D3. Stereotypy of speech | 11 | 11.1 |
|  |  |  |  |
| E. Hyperorality and dietary changes [one of the following symptoms | E1. Altered food preferences | 12 | 12.1 – 12.2 |
|  | E2. Binge eating, increased consumption of alcohol or cigarettes | 13, 14 |  |
|  | E3. Oral exploration or consumption of inedible objects | 15 |  |
|  |  |  |  |
| Additional questions | Other behavioural disorders | 16 |  |
|  | First symptom(s) and time of their start | 17 |  |

* “early” refers to symptom presentation within the first three years

In the following, you find the list of questions and items of the BDQ in German (Table A5) and in English (Table A6).

**Table A5.** *Behavioural Dysfunction Questionnaire (German version)*

| **1. Zeigt sie/er sozial unangebrachtes Verhalten, wie zum Beispiel:** |
| --- |
| 1.1. Unangemessene Annäherungen, wie fremde Personen anfassen oder körperlich ganz nahe kommen |
| 1.2. Berühren oder Küssen von fremden Personen |
| 1.3. Verbale Aggressionen, wie beschuldigen, anschreien, usw.  1.4. Körperliche Aggressionen, wie schlagen, stossen, kratzen, usw.  *1.5. Sich in der Öffentlichkeit entkleiden **  1.6. Urinieren in der Öffentlichkeit  *1.7. Unangemessene sexuelle Handlungen, wie sich in der Öffentlichkeit selbst befriedigen / masturbieren **  1.8. Kriminelles Verhalten (wie z.B. Diebstahl) |
| **2. Ist Ihnen bei ihr/ihm der Verlust von Umgangsformen oder des Anstands aufgefallen, wie zum Beispiel:** |
| 2.1. Unpassendes Lachen |
| 2.2. Fluchen oder Schreien |
| 2.3. Beleidigungen |
| 2.4. Unhöfliche oder sexuell anzügliche Bemerkungen |
| 2.5. Mangel an Anstand (z.B. nicht in der Warteschlange anstehen können) |
| 2.6. Fehlende Achtung der Privatsphäre |
| 2.7. Keine angemessene Reaktion auf soziale Signale (z.B. die Person redet weiter, obwohl ihr/ihm signalisiert wird aufzuhören) |
| 2.8. Mangelnde Körperpflege (z.B. sie/er trägt übelriechende, schmutzige, verschlissene oder unpassende Kleidung) |
| 2.9. Unhöfliches Benehmen in der Öffentlichkeit, wie furzen, an den Geschlechtsteilen kratzen, in den Zähnen herumstochern, spucken oder rülpsen |
| **3. Zeigt sie/er impulsive, unbedachte oder achtlose Handlungen, wie zum Beispiel:** |
| 3.1. Rücksichtsloses (Auto-)Fahren |
| *3.2. Neu begonnenes Glücksspiel ** |
| *3.3. Stehlen von Nahrungsmitteln oder glänzenden Objekten ** |
| 3.4. Unbedachtes Kaufen oder Verkaufen von Objekten |
| 3.5. Unbedachtes Preisgeben von persönlichen Daten wie z.B. die Nummer der Kreditkarte |
| **4. Haben Sie bemerkt, dass ihr/ihm der Antrieb fehlt, wie zum Beispiel:** |
| 4.1. Mangel an Spontanität |
| 4.2. Vermindertes oder fehlendes Interesse für Tätigkeiten, die ihr/ihm früher wichtig waren |
| **5. Ist Ihnen bei ihr/ihm träges Verhalten aufgefallen, wie zum Beispiel:**  5.1. Aufforderungen sind notwendig, damit alltägliche Verrichtungen (wie z.B. Zähneputzen) begonnen oder ausgeführt werden |
| 5.2. Gespräche werden nicht begonnen oder aufrechterhalten  **6. Ist Ihnen aufgefallen, dass sie/er weniger auf die Bedürfnisse und Gefühle anderer eingeht, wie zum Beispiel:** |
| 6.1. Gleichgültigkeit gegenüber Schmerz oder Leid anderer |
| 6.2. Verletzende Bemerkungen zum Schmerz oder Leid anderer Personen |
| **7. Ist Ihnen bei ihr/ihm ein vermindertes Interesse an sozialen Kontakten und Beziehungen aufgefallen, wie zum Beispiel:** |
| 7.1. Vermindertes Interesse an der Gesellschaft anderer |
| 7.2. Vermeiden von Blickkontakt |
| 7.3. Abnahme von sozialem Engagement  **8. Ist Ihnen bei ihr/ihm eine Abnahme von Wärme im zwischenmenschlichen Umgang aufgefallen, wie zum Beispiel:** |
| 8.1. Vermeiden von körperlichem Kontakt wie z.B. Berührung oder Umarmung von Freunden und Verwandten |
| 8.2. Emotionale Distanziertheit, d. h. nicht mehr auf äussere Einflüsse positiver oder negativer Art emotional reagieren |
| 8.3. Gefühlskälte |
| **9. Haben Sie beobachtet, dass sie/er wiederholt die gleichen Bewegungen ausführt, wie zum Beispiel:**  9.1. Reiben der Hände |
| 9.2. Klopfen mit Händen oder Füssen |
| 9.3. Klatschen der Hände |
| 9.4. Sich kratzen |
| 9.5. Sich an Haut oder Kleidern zupfen |
| 9.6. Summen |
| *9.7. Mit dem Stuhl schaukeln ** |
| 9.8. Räuspern  9.9. Mit den Lippen schmatzen |
| **10. Ist Ihnen bei ihr/ihm ein zwanghaftes oder ritualisiertes Verhalten aufgefallen, wie zum Beispiel:** |
| 10.1. Zwanghaftes Zählen  10.2. Zwanghafte Reinigungsrituale |
| 10.3. Zwanghaftes Sammeln oder Horten |
| 10.4. Zwanghaftes Kontrollieren |
| 10.5. Zwanghaftes Auf-die-Toilette-Gehen |
| 10.6. Zwanghaftes Anordnen von Gegenständen |
| 10.7. Zwanghaftes Gehen bestimmter Strecken |
| **11. Haben Sie bei ihr/ihm wiederholt sprachliche Auffälligkeiten bemerkt, wie zum Beispiel:** |
| 11.1. Zwanghaftes Wiederholen von Wörtern, Sätzen oder Erzählungen |
| **12. Sind Ihnen bei ihr/ihm veränderte Vorlieben für Nahrungsmittel aufgefallen, wie zum Beispiel:** |
| 12.1. Verstärktes Verlangen nach Süssigkeiten |
| 12.2. Einschränkung auf den Konsum bestimmter Nahrungsmittel |
| **13. Sind Ihnen bei ihr/ihm Essanfälle aufgefallen?** |
| **14. Hat sie/er neu mit dem Konsum von Zigaretten oder Alkohol begonnen, oder den Konsum von Zigaretten oder Alkohol erhöht?** |
| ***15. Haben Sie bei ihr/ihm beobachtet, dass sie/er nicht essbare Gegenstände in den Mund nimmt oder isst? **** |
| **16. Falls Ihnen Verhaltensauffälligkeiten bei Ihrer/Ihrem Angehörigen aufgefallen sind, die wir nicht erfragt haben, bitten wir Sie, diese hier anzugeben** |
| **17. Seit wann besteht eine Veränderung – sei es in der Kraft/Motorik (z.B. Gehen oder Koordination), im Denken oder Sprechen, in der Stimmung, im Verhalten oder anderweitig – bei Ihrer/Ihrem Angehörigen?**  **Welche Form der Veränderung war dies?** |

* excluded items (affirmed by less than 5% of the informants of patients with behavioural variant frontotemporal dementia)

**Table A6.** *Behavioural Dysfunction Questionnaire (English version)*

| **1. Does she/he show socially inappropriate behaviour, such as:** |
| --- |
| 1.1. Inappropriate approaches, such as touching strangers or getting very close physically |
| 1.2. Touching or kissing strangers |
| 1.3. Verbal aggression, such as blaming, yelling at, etc.  1.4. Physical aggression, such as hitting, pushing, scratching, etc.  *1.5. Undressing in public **  1.6. Urinating in public  *1.7. Inappropriate sexual acts, such as pleasuring oneself / masturbating in public **  1.8. Criminal behaviour (such as stealing) |
| **2. Have you noticed in her/him a loss of manners or etiquette, such as:** |
| 2.1. Inappropriate laughter |
| 2.2. Swearing or yelling |
| 2.3. Offensive comments |
| 2.4. Rude or sexually suggestive comments |
| 2.5. Lack of etiquette (e.g., not being able to wait in line) |
| 2.6. Lack of respect for privacy |
| 2.7. Failure to respond appropriately to social cues (e.g., continuing to talk even though it was signalled to stop) |
| 2.8. Lack of personal hygiene (e.g., she/he wears malodorous, stained, torn or inappropriate clothing) |
| 2.9. Rude behaviour in public, such as farting, scratching private parts, picking teeth, spitting, or belching |
| **3. Does she/he show impulsive, thoughtless, or careless actions, such as:** |
| 3.1. Reckless (car-)driving |
| *3.2. Newly started gambling ** |
| *3.3. Stealing food or shiny objects ** |
| 3.4. Unwise buying or selling of objects |
| 3.5. Careless disclosure of personal data such as credit card number |
| **4. Have you noticed that she/he lacks drive, such as:** |
| 4.1. Lack of spontaneity |
| 4.2. Decreased or lack of interest in activities that used to be important to her/him |
| **5. Have you noticed any sluggish behaviour in her/him, such as:**  5.1. Prompts are necessary for everyday tasks (such as brushing teeth) to be started or performed |
| 5.2. Conversations are not initiated or maintained  **6. Have you noticed that she/he is less responsive to the needs and feelings of others, such as:** |
| 6.1. Ignorance of pain or suffering of others |
| 6.2. Making hurtful comments leading to other person’s pain or suffering |
| **7. Have you noticed in her/him a decreased interest in social contacts and relationships, such as:** |
| 7.1. Decreased interest in the company of others |
| 7.2. Avoiding eye contact |
| 7.3. Decrease in social engagement  **8. Have you noticed in her/him a decreased warmth in interpersonal interactions, such as:** |
| 8.1. Avoiding physical contact, such as touching or hugging friends and relatives |
| 8.2. Emotional detachment, i.e. no longer reacting emotionally to external stimuli of a positive or negative nature |
| 8.3. Emotional coldness |
| **9. Have you observed that she/he repeatedly performs the same movements, such as:**  9.1. Rubbing hands |
| 9.2. Tapping with hands or feet |
| 9.3. Clapping hands |
| 9.4. Scratching oneself |
| 9.5. Tugging at skin or clothes |
| 9.6. Humming |
| *9.7. Rocking with chair ** |
| 9.8. Clearing throat  9.9. Smacking of lips |
| **10. Have you noticed any compulsive or ritualistic behaviours in her/him, such as:** |
| 10.1. Compulsive counting  10.2. Compulsive cleaning rituals |
| 10.3. Compulsive collecting or hoarding |
| 10.4. Compulsive controlling |
| 10.5. Compulsive going to the toilet |
| 10.6. Compulsive arrangement of objects |
| 10.7. Compulsive walking of certain routes |
| **11. Have you noticed any repeatedly language abnormalities in her/him, such as:** |
| 11.1. Compulsive repetition of words, phrases, or narratives |
| **12. Have you noticed any changes in her/his food preferences, such as:** |
| 12.1. Increased craving for sweets |
| 12.2. Restriction on consumption of certain foods |
| **13. Have you noticed her/him having any binge eating episodes?** |
| **14. Has she/he newly started consuming cigarettes or alcohol, or increased the usual consumption of cigarettes or alcohol?** |
| ***15. Have you observed her/him putting non-edible items in the mouth or eating them?**** |
| **16. If you have noticed behavioural abnormalities in your loved one that we have not asked about, please indicate them here** |
| **17. When have the first changes in your loved one started (whether in strength / motor function [e.g., walking or coordination], thinking or speaking, mood, behaviour, or otherwise)?**  **What kind of changes did you observe?** |

* excluded items (affirmed by less than 5% of the informants of patients with behavioural variant frontotemporal dementia)

# Supplementary B. Inclusion and exclusion criteria of patients and healthy participants

Patients with behavioural variant frontotemporal dementia (bvFTD) and Alzheimer's disease dementia (ADD) were recruited from two Swiss (i.e., Memory Clinic, University Department of Geriatric Medicine FELIX PLATTER, Basel; Clinic of Neurology and Neurophysiology, Canton Hospital St. Gallen) and three German (i.e., Clinic for Neurology, Münster University Hospital; Clinic for Cognitive Neurology, University Hospital Leipzig; Department of Neurology, University of Ulm) memory clinics. The diagnosis was established by a multidisciplinary team consisting of neurologists, neuropsychologists, and psychiatrists, who performed comprehensive neuropsychological and neuroimaging assessments. Inclusion criteria were the diagnosis of at least probable bvFTD [7] or probable ADD [5], and availability of a reliable informant, who has regular contact with the patient. Exclusion criteria were major neurocognitive disorder at moderate or severe stage according to DSM-5, history of or current drug and/or alcohol abuse as well as drug- and/or alcohol-related disorder according to ICD-10, history of severe depressive episode or current depressive episode according to ICD-10, history of or current major psychiatric disorders according to ICD-10, traumatic brain injury, systemic disorders or brain diseases that could result in behavioural changes.

Patients with major depressive disorder (MDD) were recruited from three Swiss institutions (i.e., Memory Clinic, University Department of Geriatric Medicine FELIX PLATTER, Basel; Clienia Schlössli AG, Clinic of Psychiatry and Psychotherapy, Oetwil am See; University Psychiatric Clinic, Basel). The inclusion criterion was the diagnosis of an at least moderate depressive episode according to ICD-10. Exclusion criteria were a neurocognitive disorder according to DSM-5, history of or current drug or/and alcohol abuse as well as drug- and alcohol-related disorder according to ICD-10, any other major psychiatric disorders according to ICD-10, traumatic brain injury, systemic disorders or brain diseases that could result in behavioural changes.

Healthy participants were recruited from the participant pool of the Memory Clinic, University Department of Geriatric Medicine FELIX PLATTER Basel, Switzerland. Inclusion criteria were at least seven years of education, German and/or Swiss German as mother tongue and self-report of good health. Exclusion criteria were conditions with potential negative influence on the behaviour, including cognitive deficits [i.e., Montreal Cognitive Assessment [6] score below the demographically-adjusted fifth percentile for cognitively healthy individuals [8]], systemic or brain diseases, psychiatric disorders according the ICD-10, traumatic brain injury, chronic pain, history of or regular intake of any psychoactive drugs (except benzodiazepines for sleep) and severe sensory and/or motor deficits. Additionally, participants were checked for signs of depressive mood by use of the Beck Depression Inventory (score ≥ ten points) [1] or the Geriatric Depression Scale (score ≥ five points) [9].

# Supplementary C. Item scores of the Behavioural Dysfunction Questionnaire of healthy participants

We analysed the item scores of the 414 healthy subjects (Table C1). Of the 56 items, four items (1.5, 1.7, 3.2, and 3.3) were never affirmed by healthy participants’ informants. Items were generally (95.2%) negated. Items were affirmed in 3% as “very mild” or “rare”, in 0.9% as “mild” or “occasionally”; in 0.5% as “moderate” or “sometimes”; in 0.3% as “severe” or “often” and in 0.1% as “very severe” or “very often”.

**Table C1.** *Answer pattern (in percentages) of informants of healthy participants*

|  | **Item score** | | | | | |  |
| --- | --- | --- | --- | --- | --- | --- | --- |
|  | **0** | **1** | **2** | **3** | **4** | **5** | **99-%*** |
| *Domain A. Early behavioural disinhibition* | | | | | | | |
| **Item 1.1** | 98.79 | 0.97 | 0 | 0 | 0.24 | 0 | 1 |
| **Item 1.2** | 98.06 | 1.94 | 0 | 0 | 0 | 0 | 1 |
| **Item 1.3** | 85.23 | 10.65 | 1.94 | 1.45 | 0.73 | 0 | 3 |
| **Item 1.4** | 99.27 | 0.48 | 0 | 0.24 | 0 | 0 | 0 |
| **Item 1.5** | 100 | 0 | 0 | 0 | 0 | 0 | 0 |
| **Item 1.6** | 99.76 | 0.24 | 0 | 0 | 0 | 0 | 0 |
| **Item 1.7** | 100 | 0 | 0 | 0 | 0 | 0 | 0 |
| **Item 1.8** | 98.79 | 1.21 | 0 | 0 | 0 | 0 | 1 |
| **Item 2.1** | 94.19 | 5.57 | 0.24 | 0 | 0 | 0 | 1 |
| **Item 2.2** | 88.35 | 7.28 | 2.91 | 0.49 | 0.73 | 0.24 | 3 |
| **Item 2.3** | 91.77 | 5.57 | 1.21 | 0.48 | 0.97 | 0 | 3 |
| **Item 2.4** | 97.82 | 1.21 | 0.73 | 0.24 | 0 | 0 | 1 |
| **Item 2.5** | 92.98 | 5.33 | 1.21 | 0.24 | 0.24 | 0 | 2 |
| **Item 2.6** | 96.13 | 3.63 | 0.24 | 0 | 0 | 0 | 1 |
| **Item 2.7** | 87.62 | 8.74 | 1.21 | 1.7 | 0.73 | 0 | 3 |
| **Item 2.8** | 97.82 | 1.7 | 0.24 | 0.24 | 0 | 0 | 1 |
| **Item 2.9** | 97.09 | 1.94 | 0.24 | 0.24 | 0 | 0.49 | 1 |
| **Item 3.1** | 94.4 | 4.14 | 0.73 | 0.49 | 0.24 | 0 | 2 |
| **Item 3.2** | 100 | 0 | 0 | 0 | 0 | 0 | 0 |
| **Item 3.3** | 100 | 0 | 0 | 0 | 0 | 0 | 0 |
| **Item 3.4** | 95.41 | 3.62 | 0.97 | 0 | 0 | 0 | 1 |
| **Item 3.5** | 99.03 | 0.97 | 0 | 0 | 0 | 0 | 0 |

| *Domain B. Early apathy or inertia* | | | | | | | |
| --- | --- | --- | --- | --- | --- | --- | --- |
| **Item 4.1** | 81.55 | 9.95 | 5.58 | 2.91 | 0 | 0 | 3 |
| **Item 4.2** | 86.96 | 9.18 | 2.66 | 1.21 | 0 | 0 | 3 |
| **Item 5.1** | 97.58 | 1.69 | 0.24 | 0.48 | 0 | 0 | 1 |
| **Item 5.2** | 90.58 | 6.04 | 1.69 | 0.97 | 0.24 | 0.48 | 3 |
| *Domain C. Early loss of sympathy or empathy* | | | | | | | |
| **Item 6.1** | 94.19 | 3.87 | 1.69 | 0.24 | 0 | 0 | 2 |
| **Item 6.2** | 95.64 | 3.39 | 0.73 | 0.24 | 0 | 0 | 1 |
| **Item 7.1** | 87.68 | 7.25 | 3.86 | 0.48 | 0.72 | 0 | 3 |
| **Item 7.2** | 94.2 | 4.35 | 0.97 | 0.24 | 0 | 0.24 | 2 |
| **Item 7.3** | 92.27 | 5.56 | 1.69 | 0.48 | 0 | 0 | 2 |
| **Item 8.1** | 94.9 | 3.16 | 0.24 | 1.21 | 0 | 0.49 | 3 |
| **Item 8.2** | 94.43 | 3.39 | 1.45 | 0.48 | 0.24 | 0 | 2 |
| **Item 8.3** | 93.69 | 4.61 | 0.97 | 0.49 | 0.24 | 0 | 2 |
| *Domain D. Early perseverative, stereotyped or compulsive/ritualistic behaviour* | | | | | | | |
| **Item 9.1** | 96.36 | 1.21 | 0 | 1.21 | 0.73 | 0.49 | 4 |
| **Item 9.2** | 96.36 | 2.18 | 0 | 0.49 | 0.73 | 0.24 | 3 |
| **Item 9.3** | 98.54 | 0.73 | 0.24 | 0.49 | 0 | 0 | 1 |
| **Item 9.4** | 94.9 | 2.18 | 0.49 | 1.21 | 0.49 | 0.73 | 4 |
| **Item 9.5** | 97.57 | 1.21 | 0.24 | 0.49 | 0.24 | 0.24 | 2 |
| **Item 9.6** | 95.87 | 2.43 | 0.97 | 0.24 | 0.24 | 0.24 | 2 |
| **Item 9.7** | 98.79 | 0.97 | 0 | 0.24 | 0 | 0 | 1 |
| **Item 9.8** | 93.19 | 3.16 | 0.97 | 0.97 | 1.22 | 0.49 | 4 |
| **Item 9.9** | 99.27 | 0.24 | 0 | 0.24 | 0.24 | 0 | 0 |
| **Item 10.1** | 98.31 | 0.48 | 0.72 | 0.48 | 0 | 0 | 2 |
| **Item 10.2** | 97.34 | 1.21 | 0.48 | 0.24 | 0.72 | 0 | 2 |
| **Item 10.3** | 93.72 | 2.9 | 0.97 | 1.45 | 0.72 | 0.24 | 3 |
| **Item 10.4** | 93.24 | 3.14 | 1.69 | 1.45 | 0.48 | 0 | 3 |
| **Item 10.5** | 98.31 | 0.72 | 0.24 | 0.48 | 0 | 0.24 | 1 |
| **Item 10.6** | 96.38 | 1.93 | 0.72 | 0.72 | 0.24 | 0 | 2 |
| **Item 10.7** | 99.52 | 0.24 | 0 | 0.24 | 0 | 0 | 0 |
| **Item 11.1** | 95.41 | 1.69 | 1.21 | 0.72 | 0.72 | 0.24 | 3 |
| *Domain E. Hyperorality and dietary changes* | | | | | | | |
| **Item 12.1** | 87.44 | 3.62 | 2.42 | 2.42 | 2.42 | 1.69 | 5 |
| **Item 12.2** | 93.24 | 2.66 | 0.97 | 0.97 | 0.97 | 1.21 | 5 |
| **Item 13** | 93.95 | 4.6 | 1.45 | 0 | 0 | 0 | 2 |
| **Item 14** | 96.13 | 1.94 | 0.48 | 0.48 | 0.73 | 0.24 | 3 |
| **Item 15** | 99.27 | 0.48 | 0.24 | 0 | 0 | 0 | 0 |

* 99-% refers to the 99-percentile of the respective item score. 0 = no; 1 = very mild / rare (less than once a month); 2 = mild / occasionally (approximately once a month but not weekly); 3 = moderate / sometimes (about once a week); 4 = severe / often (several times a week but not daily); 5 = very severe / very often (daily).

We analysed the answer pattern of healthy participants’ informants to calculate the BDQ-Global Domain Score (BDQ-GDS). BDQ-GDS represents the number of affirmed behavioural domains (0-5). According to Rascovsky, et al. [7], a domain can be considered as present if at least one of the behavioural symptoms (i.e., items) is “persistent or recurrent rather than single or rare event”. In the BDQ, this corresponds to an item score ≥ 3 (moderate / sometimes). However, as several items were affirmed with the scores greater than three also for healthy participants, we decided to add “greater than the 99-percentile in healthy subjects” as an additional criterion for domain affirmation. This later criterion was applied in 19 of the 50 items (38%). For example, item 1.3 is only considered as pathological in case of a score ≥4 (Table C1). Notably, 99-percentile of two items (i.e., 12.1, 12.2), both from the domain “Hyperorality and dietary changes”, reached a score of five.

# Supplementary D. Internal consistency of the Behavioural Dysfunctional Questionnaire

We analysed the internal consistency of the BDQ in the patients’ sample (*N* = 131) by use of the Kuder-Richardson Formula 20 (KR-20). We used KR-20, because items scores showed a strong positive skewness.

The five domains overall showed an excellent internal consistency of .92 (Table D1). Three of the five domains (“early behavioural disinhibition”, “early loss of sympathy/empathy” and “early perseverative/stereotyped behaviour”) showed good internal consistencies (α = .76 to .86). The domain “early apathy/inertia” had an acceptable (α = .67) consistency and the domain “hyperorality and dietary changes” had a poor internal consistency (α = .54). A major reason for the low internal consistencies of these two domains is their small number of items (i.e., four items). In addition, the poor internal consistency of domain “hyperorality and dietary changes” reflects its quite different behavioural disorders [altered food preferences (2 items); binge eating (1 item); increased consumption of alcohol or cigarettes (1 item)].

**Table D1.** *Internal consistencies of the Behavioural Dysfunctional Questionnaire at domain and global level in the patients’ sample*

| **Domains** | **KR-20** | **Number of items** |
| --- | --- | --- |
| Early behavioural disinhibition | .86 | 18 |
| Early apathy / inertia | .67 | 4 |
| Early loss of sympathy / empathy | .84 | 8 |
| Early perseverative, stereotyped or compulsive behaviour | .76 | 16 |
| Hyperorality and dietary changes | .54 | 4 |
| Global (all domains) | .92 | 50 |

KR-20 = Kuder-Richardson Formula 20

# Supplementary E. Discriminatory power of the domain scores and the BDQ-Global Score between bvFTD and Non-bvFTD patients

To examine whether behavioural domain scores differ in their discriminatory power between bvFTD and Non-bvFTD patients, we run univariate logistic regression with each domain mean score, followed by ROC analyses. All domain mean scores separated the two groups acceptable to excellent [3] with AUC ranging between 77.27% (early loss of sympathy/empathy) and 84.46% (early behavioural disinhibition) (Table E1). Using the Delong’s method [2], we found similar discriminatory power across the domain scores. Likewise, the BDQ-Global Score separated the two groups similarly to three of the five domain scores (i.e., early behavioural disinhibition, early perseverative, stereotyped or compulsive behaviour, and hyperorality/dietary changes). In contrast, the BDQ-Global Score separated the two groups better than the domain score of “early apathy/inertia” and the domain score of “early loss of sympathy/empathy” (p<.05).

**Table E1.** *Discriminatory power of each domain score and the BDQ-Global Score between bvFTD and Non-bvFTD patients*

| **Domains** | **Area under the curve** |
| --- | --- |
| Early behavioural disinhibition | 84.46 (CI: 77.07–91.85) |
| Early apathy / inertia | 79.61 (CI: 70.47–88.75) |
| Early loss of sympathy / empathy | 77.27 (CI: 67.72–86.83) |
| Early perseverative, stereotyped or compulsive behaviour | 80.91 (CI: 71.42–90.4) |
| Hyperorality and dietary changes | 81.5 (CI: 72.78–90.23) |
| BDQ-Global Score | 85.98 (CI: 78.73–93.22) |

CI = 95% confidence interval

# References

1. Beck AT, Ward CH, Mendelson M, Mock J, Erbaugh J (1961) An inventory for measuring depression. Archives of general psychiatry 4:561-571

2. DeLong ER, DeLong DM, Clarke-Pearson DL (1988) Comparing the areas under two or more correlated receiver operating characteristic curves: a nonparametric approach. Biometrics 44:837-845

3. Hosmer Jr. DW, Lemeshow S, Sturdivant RX (2013) Applied Logistic Regression, Third Edition. John Wiley & Sons, Inc.

4. Kertesz A, Davidson W, Fox H (1997) Frontal behavioral inventory: diagnostic criteria for frontal lobe dementi. Canadian Journal of Neurological Sciences 24:29-36

5. McKhann GM, Knopman DS, Chertkow H, Hyman BT, Jack CR, Jr., Kawas CH, Klunk WE, Koroshetz WJ, Manly JJ, Mayeux R, Mohs RC, Morris JC, Rossor MN, Scheltens P, Carrillo MC, Thies B, Weintraub S, Phelps CH (2011) The diagnosis of dementia due to Alzheimer's disease: recommendations from the National Institute on Aging-Alzheimer's Association workgroups on diagnostic guidelines for Alzheimer's disease. Alzheimer's & dementia : the journal of the Alzheimer's Association 7:263-269

6. Nasreddine ZS, Phillips NA, Bédirian V, Charbonneau S, Whitehead V, Collin I, Cummings JL, Chertkow H (2005) The Montreal Cognitive Assessment, MoCA: a brief screening tool for mild cognitive impairment. Journal of the American Geriatrics Society 53:695-699

7. Rascovsky K, Hodges JR, Knopman D, Mendez MF, Kramer JH, Neuhaus J, Van Swieten JC, Seelaar H, Dopper EG, Onyike CU (2011) Sensitivity of revised diagnostic criteria for the behavioural variant of frontotemporal dementia. Brain 134:2456-2477

8. Thomann AE, Goettel N, Monsch RJ, Berres M, Jahn T, Steiner LA, Monsch AU (2018) The Montreal Cognitive Assessment: normative data from a German-speaking cohort and comparison with international normative samples. Journal of Alzheimer's Disease 64:643-655

9. Yesavage JA, Sheikh JI (1986) 9/Geriatric depression scale (GDS) recent evidence and development of a shorter version. Clinical gerontologist 5:165-173
